# Late Life Supplementation of 25‐Hydroxycholesterol Reduces Aortic Stiffness and Cellular Senescence in Mice

**DOI:** 10.1111/acel.70118

**Published:** 2025-05-22

**Authors:** Sophia A. Mahoney, Mary A. Darrah, Ravinandan Venkatasubramanian, Serban Ciotlos, Matthew J. Rossman, Judith Campisi, Douglas R. Seals, Simon Melov, Zachary S. Clayton

**Affiliations:** ^1^ Department of Integrative Physiology University of Colorado Boulder Boulder Colorado USA; ^2^ Buck Institute for Research on Aging Novato California USA; ^3^ Lawrence Berkeley National Laboratory Berkeley California USA

**Keywords:** aging, cellular senescence, elasticity, p16, replicative senescence

## Abstract

Stiffening of the aorta is a key antecedent to cardiovascular diseases (CVD) with aging. Age‐related aortic stiffening is driven, in part, by cellular senescence—a hallmark of aging defined primarily by irreversible cell cycle arrest. In this study, we assessed the efficacy of 25‐hydroxycholesterol (25HC), an endogenous cholesterol metabolite, as a naturally occurring senolytic to reverse vascular cell senescence and reduce aortic stiffness in old mice. Old (22–26 months) p16‐3MR mice, a transgenic model allowing for genetic clearance of p16‐positive senescent cells with ganciclovir (GCV), were administered vehicle, 25HC, or GCV to compare the efficacy of the experimental 25HC senolytic versus genetic clearance of senescent cells. We found that short‐term (5d) treatment with 25HC reduced aortic stiffness in vivo, assessed via aortic pulse wave velocity (*p* = 0.002) to a similar extent as GCV. Ex vivo 25HC exposure of aorta rings from the old p16‐3MR GCV‐treated mice did not further reduce elastic modulus (measure of intrinsic mechanical stiffness), demonstrating that 25HC elicited its beneficial effects on aortic stiffness, in part, through the suppression of excess senescent cells. Improvements in aortic stiffness with 25HC were accompanied by favorable remodeling of structural components of the vascular wall (e.g., lower collagen‐1 abundance and higher α‐elastin content) to a similar extent as GCV. Moreover, 25HC suppressed its putative molecular target CRYAB, modulated CRYAB‐regulated senescent cell anti‐apoptotic pathways, and reduced markers of cellular senescence. The findings from this study identify 25HC as a potential therapy to target vascular cell senescence and reduce age‐related aortic stiffness.

## Graphical abstract

25‐hydroxycholesterol (25HC) targets senescent cell anti‐apoptotic pathways (SCAPs) to reduce vascular cell senescence, favorably remodel aortic wall components, and reduce aortic stiffening with advancing age.

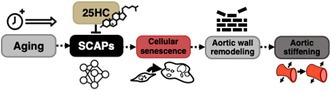

Cardiovascular diseases (CVD) remain the leading cause of morbidity and mortality worldwide (Martin et al. 2024). Aging is the primary risk factor for CVD development, which is preceded by the stiffening of the aorta (Chirinos et al. 2019). Aortic stiffness is an independent predictor of future CVD and serves as a therapeutic target to mitigate CVD risk with aging (Chirinos et al. 2019).

A fundamental aging process that contributes to aortic stiffness and CVD is cellular senescence, a multi‐factorial stress response that is characterized, in part, by cell cycle arrest (Clayton et al. 2023; Demaria et al. 2014). Senescent cell burden increases with advancing age and contributes to aortic stiffening by promoting chronic inflammation, excess oxidative stress, and adverse structural alterations that confer stiffening (i.e., α‐elastin degradation and collagen‐1 deposition) (Clayton et al. 2023; Ungvari et al. 2018). Previous studies have demonstrated that systemic clearance of senescent cells in old mice with senolytics reduces aortic stiffness, in part, by favorably modulating structural components of the vascular wall (Clayton et al. 2023; Mahoney et al. 2023). Given that senescent cell burden accumulates in various cell types and through varied mechanisms (Campisi 2013), it is important to develop novel, naturally occurring senolytic compounds that can target cellular senescence to mitigate age‐related aortic stiffening.

25‐Hydroxycholesterol (25HC) is an endogenous metabolite of cholesterol biosynthesis that has emerged as a potent senolytic therapy in skeletal muscle of old mice (Limbad et al. 2022). 25HC regulates various biological processes including antiviral activity, inflammation, and cholesterol metabolism (Cao et al. 2020). In states of excessive cellular senescence, 25HC targets the small heat shock protein αB‐crystallin (CRYAB) (Limbad et al. 2022), which is upregulated in senescent cells and activates senescent cell anti‐apoptotic pathways (SCAPs) that confer cell cycle arrest by preventing apoptosis (Demaria et al. 2014). As such, 25HC may permit apoptosis to resume in senescent cells with high CRYAB expression, as previously demonstrated in skeletal muscle cells (Limbad et al. 2022). Given that the vascular smooth muscle is a predominant component of the aorta, we hypothesized that 25HC supplementation would reduce the burden of aortic senescent cells and lower aortic stiffness in old mice.

## 1 Animals

**FIGURE 1:**
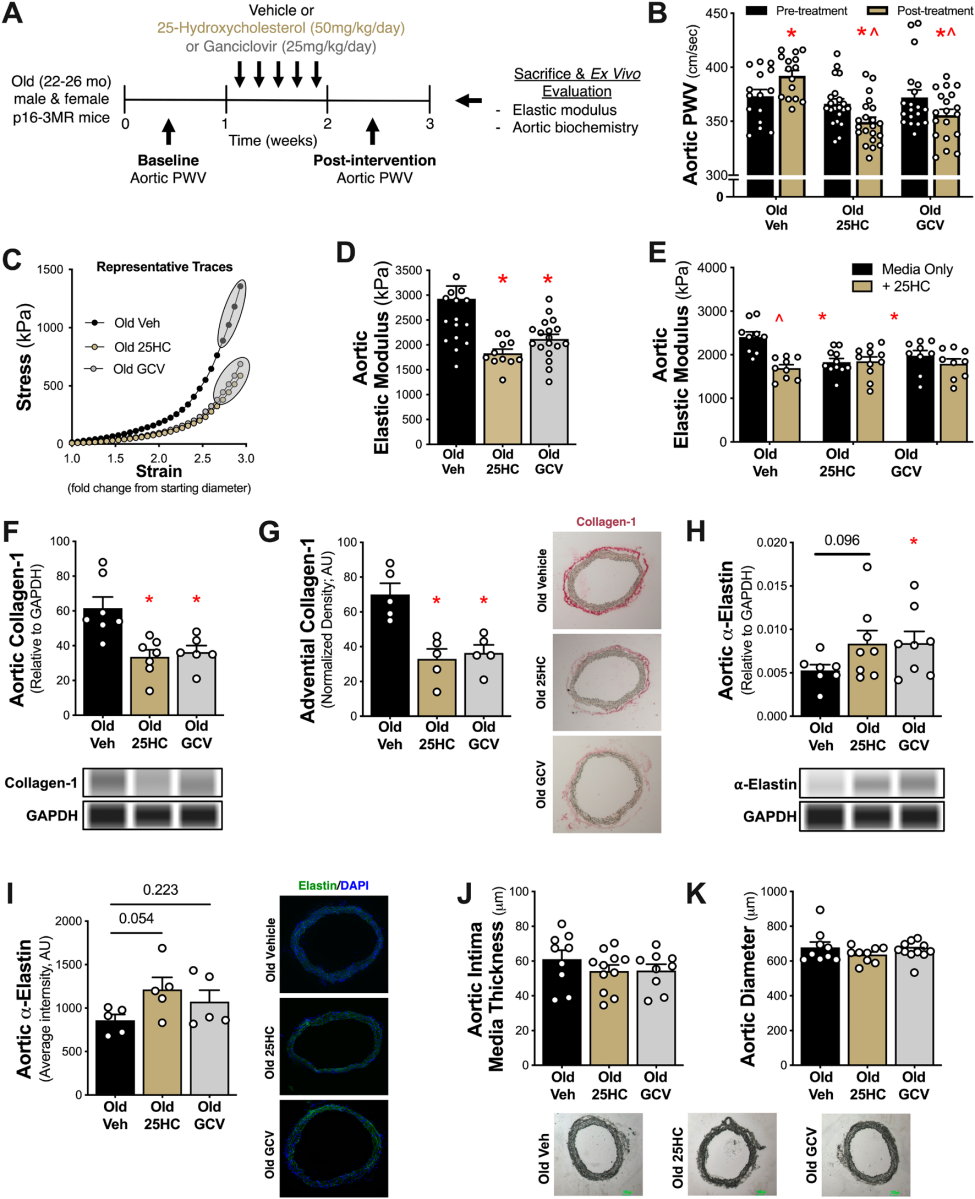
25‐Hydroxycholesterol (25HC) lowers aortic stiffness in old mice by targeting vascular cell senescence and by favorably remodeling the vascular wall. Schematic of dosing paradigm and measurements in old p16‐3MR mice (A). Aortic pulse wave velocity (PWV) pre‐ and post‐intervention (B) in old vehicle (veh), 25HC, and ganciclovir (GCV) mice. Representative traces of stress–strain curves (C) used to calculate aortic elastic modulus (D). Aortic elastic modulus assessed following 48 h incubation with media only or media with 25HC (E). Quantification of type‐1 collagen by aortic abundance (F) and adventitial immunohistochemistry abundance (with representative images) (G). Quantification of α‐elastin by aortic abundance (H) and aortic immunofluorescence (with representative images (I). Aortic intima media thickness (J) and diameter (K) with reference images. AU, Arbitrary units. Data represent mean ± SEM. *p < 0.05 vs. old veh mice. ^*p* < 0.05 vs. pre‐treatment or media only conditions.

In this study, we leveraged the transgenic p16‐3MR mouse model which allows for genetic‐based clearance of p16‐expressing senescent cells with the drug ganciclovir (GCV) (Clayton et al. 2023; Demaria et al. 2014). Old (22–26 months) male and female p16‐3MR mice were administered vehicle (veh; *n* = 19, 9F/10M), 25HC (*n* = 21, 11F/10M, 50 mg/kg/day), or GCV (*n* = 16, 9F/7M, 25 mg/kg/day) via intraperitoneal injection for 5 consecutive days. Mice were euthanized 1–2 weeks following the final dose (Figure 1A). Given that we have not previously observed sex differences in any measures of aortic stiffness or cellular senescence in p16‐3MR mice (Clayton et al. 2023; Mahoney et al. 2023), male and female data were combined.

## 2 25HC Lowers Aortic Stiffness in Old Mice by Suppressing Excess Vascular Cell Senescence

We previously established that vascular cell senescence is implicated in age‐related aortic stiffening (Clayton et al. 2023; Mahoney et al. 2023). As such, we aimed to determine whether 25HC reduces aortic stiffness in old mice. To accomplish this, we measured aortic pulse wave velocity (PWV) in vivo, the gold standard approach for assessing aortic stiffness, pre‐ and post‐intervention (Clayton et al. 2023; Mahoney et al. 2023) and then sought to compare 25HC to GCV‐based senescent cell clearance. Across all groups, there were no baseline differences in PWV prior to treatment (veh, 373 ± 6 v. 25HC, 366 ± 5 v. GCV, 372 ± 7 cm/s, *p* = 0.404). In the old veh mice, we observed a modest increase in PWV between the pre‐ to post‐intervention (post, 392 ± 5 cm/s, *p* = 0.001). 25HC reduced PWV (post, 349 ± 5 cm/s, *p* = 0.002) and 25HC mice had lower PWV relative to the veh mice (*p* < 0.001). Likewise, GCV reduced PWV (post, 356 ± 5 cm/s, *p* = 0.045), and had comparable post‐intervention PWV v. 25HC mice (*p* = 0.359) indicating similar improvements in aortic stiffness (Figure 1B).

To determine whether structural changes to the vascular wall might contribute to the observed lower aortic PWV with 25HC, we measured ex vivo aortic elastic modulus, defined by the association between the change in stress on the vascular wall with increasing strain (stretch) and indicative of the intrinsic mechanical stiffness (Figure 1C). We found that aortas from old 25HC mice had lower elastic modulus v. old veh mice (25HC, 1832 ± 83 v. veh, 2928 ± 257 kPa, *p* = 0.003). Further, old 25HC mice had similarly lower aortic elastic modulus to the GCV mice (GCV, 2118 ± 96 kPa, *p* = 0.394; Figure 1D), suggesting that 25HC and GCV reduce aortic stiffness by favorably modulating aortic intrinsic mechanical stiffness.

We next aimed to determine if 25HC had a direct effect on aortic stiffness and if these improvements were mediated by a reduction of cellular senescence. To test this, we incubated aortic rings from the treated mice with either control media or media supplemented with 1 mM 25HC for 48 h prior to assessing aortic elastic modulus. Aortas from old veh mice demonstrated reduced elastic modulus after ex vivo incubation with 25HC v. media only, indicating that 25HC acts directly on the aorta to lower intrinsic wall stiffness (with 25HC, 1691 ± 82 v. media only, 2406 ± 120 kPa, *p* < 0.001). Aortas from old 25HC mice had lower elastic modulus following media only incubation relative to old veh aortas, while there were no further improvements after ex vivo 25HC incubations (media only, 1832 ± 83 v. with 25HC, 1847 ± 106 kPa, *p* = 0.876). Finally, aortas from old GCV mice also had lower elastic modulus after media only incubation relative to old veh aortas and had no further improvements in elastic modulus after ex vivo incubation with 25HC (media only, 1977 ± 122 v. with 25HC, 1793 ± 112 kPa, *p* = 0.224; Figure 1E). Given that we did not observe further reduction in elastic modulus in aortas from old GCV mice following ex vivo 25HC incubation, this suggests that 25HC may improve intrinsic mechanical wall stiffness by reducing senescent cells and that 25HC has direct effects on the aorta to reduce stiffness.

Age‐related increases in vascular wall stiffness are mediated in part by adverse remodeling of structural components in the vascular wall, including collagen‐1 deposition and α‐elastin degradation (Chirinos et al. 2019). Collagen‐1 is the main load‐bearing element in the vascular wall and serves as a stiff reinforcing structural element. We found that old 25HC (−42%, *p* = 0.008) and GCV (−38%, *p* = 0.016) treated mice had lower aortic collagen‐1 abundance v. old veh mice (Figure 1F). Changes in collagen‐1 abundance were confirmed to be changes in the adventitia region of the aorta, increased in both 25HC (−53%, *p* = 0.001) and GCV (−49%, *p* = 0.002) mice v. old veh mice (Figure 1G). Further, aortic α‐elastin, the primary matrix protein responsible for the elasticity of arteries, was increased in both 25HC (+59%, *p* = 0.096) and GCV (+59%, *p* = 0.069) mice v. old veh mice (Figure 1H), and improvements in aortic α‐elastin were confirmed by immunofluorescence in 25HC (+41%, *p* = 0.054) and GCV (+25%, *p* = 0.223) mice v. old veh mice (Figure 1I). Importantly, these improvements occurred independently of changes in aortic intima media thickness (*p* = 0.437; Figure 1J) or diameter (*p* = 0.872; Figure 1K). Together, these findings indicate that favorable changes in structural components of the vascular wall may underlie 25HC‐mediated improvements in aortic stiffness.

## 3 25HC Targets Vascular Cell Senescence by Favorably Modulating CRYAB‐Regulated SCAPs

**FIGURE 2:**
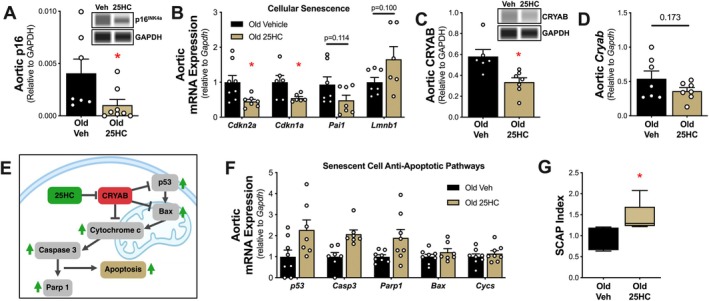
25‐Hydroxycholesterol (25HC) targets vascular CRYAB to favorably modulate senescent cell anti‐apoptotic pathways (SCAPs) and cellular senescence burden. Aortic protein abundance of cellular senescence marker p16 (A). Aortic gene expression of the cellular senescence markers (B). Aortic protein abundance (C) and gene expression (D) of CRYAB levels. Schematic of CRYAB‐regulated SCAP genes and putative effects of 25HC (E). Aortic gene expression (F) and composite index (G) of CRYAB‐regulated SCAP genes. Data represent mean ± SEM. *p < 0.05 vs. old vehicle (veh) mice.

Given that 25HC improved aortic stiffness in old mice, we next sought to characterize the effects of 25HC on vascular senescent cell burden and the associated mechanisms of action. (Baker et al. 2011). We found that 25HC mice had 75% lower aortic abundance of the cell cycle inhibitor p16 (*p* = 0.028; Figure 2A). Additionally, gene expression of cellular senescence was favorably modulated in old 25HC mice vs. old veh mice including *Cdkn2a* (−55%, *p* = 0.022), *Cdkn1a* (−45%, *p* = 0.042), *Pai1* (−52%, *p* = 0.114), and *Lmnb1* (+66%, *p* = 0.100; Figure 2B). Together, these data suggest that vascular cell senescence is reduced with 25HC supplementation.

CRYAB is a small heat shock protein that is upregulated and aggregated in states of cellular senescence and the target of 25HC (Limbad et al. 2022). To test the effects of 25HC on CRYAB in the vasculature, we assessed CRYAB abundance in aortas from old mice following 25HC supplementation. We found that old 25HC mice had 38% lower aortic CRYAB abundance vs. old veh mice (*p* = 0.028; Figure 2C). Similarly, aortic *Cryab* gene expression tended to be lower in old 25HC vs. old veh mice (−33%, *p* = 0.173; Figure 2D).

CRYAB is known to regulate SCAP signaling, which contributes to apoptosis resistance and sustained cell cycle arrest in senescent cells (Zhang et al. 2019) (Figure 2E). As such, we measured the aortic expression of CRYAB‐regulated SCAP genes and found 25HC mice had generally upregulated expression of SCAP genes, including cell cycle regulator *p53* (+156%, *p* = 0.058) and apoptosis regulators *Casp3* (+136%, *p* = 0.069), *Bax* (+71%, *p* = 0.220), *Parp1* (+65%, *p* = 0.212), and *Cycs* (+15%, *p* = 0.453) vs. old veh mice (Figure 2F). Importantly, SCAP signaling represents multiple interrelated biological pathways and functions. As such, examining CRYAB‐regulated SCAPs as a biomarker‐composite index may be more robust than examining each individual factor to distinguish their regulation. As such, we next measured a SCAP index, calculated by averaging the normalized SCAP factors per sample (Diniz et al. 2022; Seitz‐Holland et al. 2023; Suda et al. 2023), and found a 35% difference in CRYAB‐regulated SCAPs between groups (*p* = 0.010; Figure 2G), suggesting that CRYAB inhibition with 25HC favorably modulates CRYAB‐regulated aortic SCAP signaling in old mice. Mechanistically, 25HC may directly interfere with the aggregation of CRYAB proteins, leading to proteostatic stress and apoptosis (Cao et al. 2020). Further, 25HC antagonizes CRYAB‐regulated SCAP pathways, allowing apoptosis to resume in senescent cells with high CRYAB expression (Cao et al. 2020; Zhang et al. 2019).

The present study provides the first evidence that late‐life 25HC supplementation improves aortic stiffness in mice by reducing age‐related vascular cell senescence. Reductions in aortic stiffness with 25HC supplementation were mediated by senescent cell clearance and favorable vascular wall remodeling, including lower collagen‐1 abundance and higher α‐elastin content. We also demonstrate that 25HC targets CRYAB and modulates the SCAPs in the aorta. Together, this study establishes a new senolytic intervention to target age‐related aortic stiffness and poses a promising strategy to potentially translate to mid‐life and older adults to reduce CVD risk.

## Author Contributions

S.A.M., M.J.R., S.C., J.C., D.R.S., S.M., and Z.S.C. contributed to the conception, experimental design, and interpretation of the data. S.A.M., M.A.D., R.V., S.C., and Z.S.C. collected the data. S.A.M., M.A.D., R.V., S.C., M.J.R., D.R.S., S.M., and Z.S.C. were involved with the preparation and final approval of the manuscript.

## Conflicts of Interest

The authors declare no conflicts of interest.

## Supporting information


Data S1.

## Data Availability

The data that supports the findings of this study are available in the Supporting Information of this article.
