# Supplementary material for: Late Life Supplementation of 25‐Hydroxycholesterol Reduces Aortic Stiffness and Cellular Senescence in Mice
Source: Aging Cell. 2025 May 22;24(8):e70118. doi: 10.1111/acel.70118 (PMC12341771; doi:10.1111/acel.70118)
Supplement: Supplementary file 1 — Data S1. [file ACEL-24-e70118-s001.docx]

**SUPPLEMENTAL DATA**

**Table S1. Antibodies.**

| Protein | Species | Dilution factor | Company | Catalog no. |
| --- | --- | --- | --- | --- |
| Collagen-1 | Rabbit | 1:10 | Sigma Aldrich | 234167 |
| α-Elastin | Rabbit | 1:20 | Invitrogen | PA572440 |
| p16^INK4A^ | Rabbit | 1:20 | Invitrogen | PA5-20379 |
| αB-crystallin (CRYAB) | Rabbit | 1:10 | Sigma Aldrich | AV50490 |
| Glyceraldehyde 3-phosphate dehydrogenase (GAPDH) | Rabbit | 1:200 | Cell Signaling | 14C10 |

**Table S2. Primer sequences**.

| Gene | Species | Forward primer | Reverse primer |
| --- | --- | --- | --- |
| *Cdkn2a* | Mouse | CCCAACGCCCCGAACT | GCAGAAGAGCTGCTACGTGAA |
| *Cryab* | Mouse | GTTCTTCGGAGAGCACCTGTT | GAGAGTCCGGTGTCAATCCAG |
| *p53* | Mouse | ATGTTCCGGGAGCTGAATGA | GAAGTGATGGGAGCTAGCAGT |
| *Casp3* | Mouse | GCTTGGAACGGTACGCTAAG | GAGTCCACTGACTTGCTCCC |
| *Parp1* | Mouse | CGAGTGGAGTACGCGAAGAG | TCGAACATGGGTGACTGCAC |
| *Bax* | Mouse | CTCAAGGCCCTGTGCACTAAAGTG | CAGCCACCCTGGTCTTGGATC |
| *Cycs* | Mouse | ACCAGCCCGGAACGAATTAAA | CCGAACAGACCGTGGAGATT |
| *Gapdh* | Mouse | AAGGTCATCCCAGAGCTGAA | CTGCTTCACCACCTTCTTGA |

**Supplemental Methods**

***Animals and Experimental Design.***

Male and female p16-3MR mice were bred in our mouse colony at the University of Colorado Boulder. These mice carry a trimodal fusion protein (3MR) under the control of the p16^INK4A^ promoter which allows for selective genetic clearance of p16^INK4A^-positive senescent cells by administering the antiviral agent ganciclovir (GCV) (Demaria et al., 2014). For the duration of the study, all mice were single housed at the University of Colorado Boulder animal facility with a 12-hr:12-hr light-dark cycle and allowed ad libitum access to an irradiated, fixed, and open rodent chow (Inotiv/Envigo 7917, stored at room temperature). For the intervention period p16-3MR mice were randomly assigned to receive vehicle (22.5% 2-hydroxypropyl-β-cyclodextrin), 25HC (50 mg/kg/day in the vehicle) (Limbad et al., 2022), or GCV (25mg/kg/day in saline). All treatments were administered via intraperitoneal injection for 5 consecutive days and mice were sacrificed 1-2 weeks following the final dose.

***In Vivo Aortic Stiffness.***

Aortic stiffness was assessed using the reference standard non-invasive *in vivo* measure, aortic pulse wave velocity (PWV), one week before (pre) and one week after (post) the intervention, as previously described (Clayton et al., 2023; Mahoney et al., 2023). Briefly, mice were placed under light isoflurane anesthesia (1.0%–2.5%) and positioned supine on a warmed heat pad. Front- and hind-limb paws were then secured to corresponding ECG electrodes. Two Doppler probes were then placed on the skin at the transverse aortic arch and the abdominal aorta. Once clear R-waves were registered, three repeated 2-second ultrasound tracings were recorded and average pre-ejection time (i.e., time between the R-wave of the ECG to the foot of the Doppler signal) was determined for each location. To calculate aortic PWV, the distance between the two probes was divided by the difference between the transverse aortic arch and abdominal aorta pre-ejection times (time_abdominal_ – time_arch_) and is reported as centimeters/second (cm/s).

***Aortic Intrinsic Mechanical Wall Stiffness (Elastic Modulus).***

Aortas were promptly excised from the mice following sacrifice, rinsed with cold physiological saline solution, and cleared of any remnant perivascular adipose and connective tissue. To measure *ex vivo* aortic stiffness, two thoracic aorta samples (~1mm in length) were cut and used to determine intrinsic mechanical stiffness via pin myography as we have previously described (Clayton et al., 2023; Mahoney et al., 2023). In short, aorta samples were placed in heated (37°C) baths filled with calcium-free, phosphate-buffered saline (PBS). The samples were then mounted on two wire prongs, followed by three rounds of pre-stretching. Once pre-stretching was complete, aortic ring diameter was increased until 1mN of force was reached and incrementally increased by 5µm every 3 minutes thereafter until failure. The force corresponding to each stretching interval was recorded and used to calculate stress and strain. A stress-strain curve was then generated using the following equations:

where *d* is diameter and *d_i_* is initial diameter.

where *L* is one-dimensional load, *H* is intima media thickness, and *D* is vessel length.

The elastic modulus of the stress-strain curve was determined as the slope of the linear regression fit to the final four points of the stress-strain curve, as previously reported by our laboratory (Clayton et al., 2023; Mahoney et al., 2023). To assess the mechanisms underlying improvement in 25HC-mediated destiffening, aortic rings were pre-incubated in either control media (DMEM + 10% fetal calf serum + 1% penicillin/streptomycin) or control media supplemented with 1mM 25HC for 48h prior to assessing aortic elastic modulus. Aortic intima media thickness and diameter were assessed as we have described previously (Clayton et al., 2023; Mahoney et al., 2023). Briefly, aortic rings (1mm) were frozen in optimal cutting temperature solution and stored at -80°C until the time of sectioning. Aortic sectioning was performed on a cryostat (7µm; Leica CM300, Leica Biosystems, Wetzlar, Germany) at -22°C and sections were visualized, and images were captured with a bright-field microscope. Aortic intima media thickness and diameter were calculated using ImageJ software.

***Aortic Protein Abundance.***

Protein abundance was measured in segments of thoracic aorta following mechanical homogenization in radioimmunoprecipitation assay lysis buffer supplemented with protease and phosphatase inhibitors (1mM sodium orthovanadate, 1X complete mini protease inhibitor cocktail tablet [Roche, Mannheim, Germany, Cat. No. 11836153001], 1mM phenylmethylsulfonyl fluoride, 1:100 Phosphatase Inhibitor Cocktail [Sigma-Aldrich, Cat. No. P2850], 5mM sodium fluoride, and 5mM sodium pyrophosphate). Total protein content was quantified using a bicinchoninic acid assay (Thermo Fisher Scientific, Cat. No. 23225). Next, abundance of collagen-1, α-elastin, p16^INK4A^, αB-crystallin (CRYAB) and Glyceraldehyde 3-phosphate dehydrogenase (GAPDH) (antibody descriptions reported in **Table S2**) were determined by loading 20 ng/mL of aortic protein per capillary in a 25-lane (capillary) automated Western blot quantitative analyzer (WES, ProteinSimple, San Jose, CA), according to the manufacturer’s guidelines, as described previously (Clayton et al., 2023; Mahoney et al., 2023), following the validation of these antibodies in test aorta lysates. Secondary antibodies were provided by the manufacturer and used according to the manufacturer’s guidelines. A grayscale analysis of the band intensities was then performed to quantify protein abundance using Compass software (ProteinSimple), with target proteins expressed relative to a loading control (GAPDH).

***Aortic Gene Expression.***

mRNA gene expression was measured in segments of thoracic aorta following mechanical homogenization. RNA was extracted using the RNeasy mini kit (Qiagen, Hilden, Germany). cDNA was synthesized using the iScript cDNA synthesis kit (Bio-Rad Laboratories, Hercules, CA). Transcripts of cellular senescence, CRYAB, and senescent cell anti-apoptotic pathways genes (primer sequences reported in **Table S3**) were analyzed using a StepOnePlus Real-Time PCR System (Applied Biosystems, Waltham, MA) in 96-well plates and the Taqman OpenArray (Applied Biosystems, Waltham, MA) was used as a master mix, as described (Clayton et al., 2023; Mahoney et al., 2023). SimpleSeq DNA sequencing (Quintara Biosciences, Cambridge, MA) was used to validate PCR products.

***Statistical Analyses.***

Power calculations were performed using G*power 3.1 (RRID: SCR_013726) for our primary outcome variable, aortic PWV. Previously, our laboratory has obtained effect sizes of 1.35 when comparing aortic PWV between treatment groups. With this effect size, N = 6 mice per condition were required to achieve 99% statistical power. Additional mice were studied in each group to ensure sufficient PWV traces were obtained and to account for age-related attrition.

Statistical analyses were conducted using GraphPad Prism version 10.4.0 (GraphPad Software, Inc., San Diego, CA, USA; RRID:SCR_002798). Data were assessed for statistical outliers (ROUT test; Q = 1%), and outliers were excluded from final analyses. All variables were assessed using one-way ANOVA or unpaired t-test. Statistical significance was set to α=0.05. Data are presented as mean ± SEM.
